# Lightweight Natural Fiber Insulation Boards Produced with Kapok Fiber (*Ceiba Pentandra*) and Polylactic Acid or Bicomponent Fiber as a Binder

**DOI:** 10.1002/gch2.202400310

**Published:** 2025-01-27

**Authors:** Aldo Joao Cárdenas‐Oscanoa, Elmar Bonaccurso, Wolfgang Machunze, Markus Euring, Kai Zhang

**Affiliations:** ^1^ Department of Wood Technology and Wood‐Based Composites Georg‐August University Goettingen 37073 Goettingen Germany; ^2^ Forest Industry Department Faculty of Forest Sciences Universidad Nacional Agraria La Molina 15024 Lima Perú; ^3^ Airbus Central Research &Technology Willy‐Messerschimitt‐Str. 1 82024 Taufkirchen Germany

**Keywords:** bicomponent fibers, insulation boards, kapok fiber, polylactic acid (PLA)

## Abstract

Traditionally, kapok fiber is employed as filling for soft pillows, bedding, and diverse elements. Due to its buoyancy and proportion between cell wall and lumen, it is also applied as buoyant material in life vests and insulation materials. This study examine slightweight insulation panels produced from kapok fibers. Lightweight insulation boards are produced by hot‐air using kapok fibers (95%) bonded with polylactic acid or bicomponent fiber (5%), achieving very low densities of 10,15, and 20 kg.m^−3^. The technological attributes like density, porosity, water absorption, wettability, compression, and thermal conductivity, are evaluated against commercial glass wool. In terms of water absorption rates, there is a direct correlation with density. All the variables reach short‐term water absorption values less than 1 kg.m^−2^, which are comparable to commercial standards. This can be attributed to the lower density, higher porosity of the samples, and the inherent hydrophobic wax layer in the cell wall surface of kapok fibers. This trend is also evident in wettability tests, where produced boards demonstrated water‐repellency when exposed to water. Regarding the mechanical property of compression, neither the binder nor the density significantly impacts compression strength. The thermal conductivity performance of kapok‐based boards is comparable with commercially available ones.

## Graphical abstract

This research explores the creation of lightweight insulation boards through a hot‐air process, utilizing kapok fibers and polylactic acid (PLA) as a binding agent. Both components are derived from natural resources and are regarded as naturally renewable. The boards produced exhibit remarkable thermal conductivity and resistance to water absorption, making them comparable to commercial alternatives.

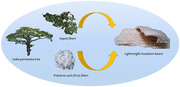

## 1 Introduction

Nowadays, there is a growing interest in the utilization of renewable and biodegradable materials as a measure to counteract climate change. Consequently, the shift from fossil‐based products to natural alternatives has become a primary focus for many researchers. Insulation boards, particularly glass wool (GW) and rockwool (RW), are among the most commonly used fossil‐based products (their production involves melting rock materials at 1500–1600 °C). These types of insulating materials, however, are known for the health risks associated with their manufacturing and use.^[^ ^1^ ^]^ They can emit minute fibers into the air, which can be harmful when inhaled (respirable mineral fibers with a diameter of <3 µm and a length of >5 µm).^[^ ^2^ ^]^ Long‐term exposure to these fibers can result in respiratory complications like throat irritation, coughing, and lung inflammation, therefore having installation difficulties due to the fibers they release. The emission of volatile organic compounds from binders, usually phenol formaldehyde resins are also a concern.^[^ ^2^ ^]^ These materials also have a substantial carbon footprint and a detrimental impact on the environment due to the high energy and resources required for their manufacture.^[^ ^1^, ^3^ ^]^ This includes greenhouse gas emissions and the disposal of solid waste. GW and RW are also non‐biodegradable, adding to landfill waste and causing prolonged environmental damage.

For these reasons, demand for natural insulation products based on natural fibers (e.g., flax, hemp, wood, wool, sheep wool, goat wool etc.) has been growing.^[^ ^4^, ^5^, ^6^, ^7^ ^]^ Natural fibers have excellent advantages over synthetic ones. They are renewable and sustainable, biodegradable, plentifully available, and they are a source that mainly comes from regular woods like hardwoods and softwoods. Nevertheless, natural fibers can be obtained also from other crops like date palm,^[^ ^8^, ^9^, ^10^ ^]^ sugar cane,^[^ ^11^ ^]^ pineapple,^[^ ^12^ ^]^ hemp,^[^ ^13^, ^14^ ^]^ kenaf,^[^ ^15^, ^16^ ^]^ kapok,^[^ ^17^, ^18^ ^]^ sunflower and watermelon seeds^[^ ^19^ ^]^ and so on. In addition, natural fibers for insulation products exhibit superior thermal characteristics, have an inherent breathability, softness and are naturally hypoallergenic, allowing an easy installation and comfort in buildings.^[^ ^5^, ^20^ ^]^ Also, natural fibers itself improve physical and mechanical properties not only by itself but for reinforcing materials, like in wood fiber insulation boards (WFIB), which are commonly used in order to avoid high and low temperatures inside constructions, generally houses and buildings.^[^ ^4^, ^6^, ^21^ ^]^

Generally, insulation boards can be categorized into two types: rigid or pressure resistant (with densities exceeding 100 kg m^−3^) and the flexible resistant ones (with densities below 100 kg m^−3^). The production of rigid wood fiber insulation boards typically involve a continuous steam process, with polymers diphenylmethandiisocyanate (pMDI) being the common binder. The flexible types usually incorporate bicomponent fibers (BF) and are processed under a hot air process. Recently, researchers developed a hot air‐hot steam process, in order to reach higher temperatures necessary for using natural binders.^[^ ^22^, ^23^ ^]^

Among natural fibers, kapok fibers are an excellent candidate as an absorbing and reinforcement material for different products,^[^ ^24^ ^]^ among them insulation boards. Sun et al.^[^ ^25^ ^]^ developed vacuum insulation panels (VIP) through a wet‐process using kapok fibers. The resulting VIP exhibited a density of 105 kg m^−3^, a porosity of 94%, and a thermal conductivity of 34.3 mW m^−1^ K^−1^ under atmospheric pressure. These characteristics, alongside a lower cost index compared to fumed silica, demonstrate the potential of kapok fibers for use in insulation materials. Nevertheless, since kapok fibers are hydrophobic, the traditional wet‐process results in loosely‐bound fibers, which reduces their longevity. To address this, Sun et al.^[^ ^26^ ^]^ incorporated lignin into the VIPs. The addition of lignin improved the mechanical strength and extended the service life. Furthermore, it decreased the thermal conductivity by 13.8% and 28% with lignin contents of 5% and 30%, respectively. Additionally, the inclusion of lignin prolonged the VIP's durability; after 28 d of accelerated weathering, the thermal conductivity increased by only 4.49 mW m^−1^ K^−1^, satisfying the criteria for commercial use.

Kapok fibers, coming from *Ceiba pentandra* seeds, are unicellular, between 10 and 25 mm in length, covered by a hydrophobic waxy layer in the cell wall which makes them very hydrophobic, therefore waterproof. Kapok fibers have a very big lumen in proportion to the cell wall, forming hollow microtubes, which contain as much as 80% air.^[^ ^18^, ^27^ ^]^ This unique characteristic makes kapok the ideal fiber for WFIB among other fibers. The seeds contain about 89% dry matter including 20–35% protein, 30% oil, and 20–26% crude fiber.^[^ ^18^ ^]^ Regarding its chemical content, kapok fiber is composed of 38.09% α‐cellulose, 14.09% lignin, and 2.34% wax content.^[^ ^27^ ^]^ The lignin fiber content of kapok fiber could reach up to 22%.^[^ ^28^ ^]^ Regarding global production, kapok raw fiber production has remained at varying levels in the world. In 2000, was 130 256 tons; in 2010, was 76 956 tons; and in 2021, it was 75 472 tons.^[^ ^29^ ^]^

Wood fiber insulation boards are commonly bound with pMDI, a binder based on non‐renewable materials. Therefore, the search for natural binders in order to reach a completely green material has been developed by different researchers from diverse origins,^[^ ^30^, ^31^ ^]^ among them lignin,^[^ ^32^, ^33^, ^34^ ^]^ canola,^[^ ^35^, ^36^, ^37^, ^38^ ^]^ starch,^[^ ^39^ ^]^ dextran^[^ ^40^ ^]^ and so on. In recent years, polylactic acid (PLA) has been emerging as a natural binding option against synthetic ones like polymeric methylene diphenil diisocyanate (pMDI), urea‐formaldehyde (UF), melamine urea‐formaldehyde (MUF), bicomponent fiber, carboxymethylcellulose sodium salt (CMC), extruded polystyrene (XPS), and polyurethane (PU).^[^ ^7^ ^]^ Kapok‐based composites with a compressed density of 1.173 kg m^−3^ with molasses (sodium silicate) as a binder were produced reaching values of thermal conductivity of 0.0220 w m^−1^ K^−1^.^[^ ^41^ ^]^

The start of PLA development can be traced back to the lactide production processes that were disclosed by Bischoff and Walden in 1893. In 1932, Carothers and his team were able to generate PLA of low molecular weight. By 1954, E.I. DuPont de Nemours and Ethicon, Inc. started to promote PLA for medical purposes such as sutures, implants, and drug‐delivery systems. Moving forward to 2002, Cargill Dow LLC in the United States began commercial production of PLA from starch, selling it under the brand name NatureWorks with an annual capacity of 140 000 tons. Finally, in 2003, the company introduced their PLA fiber, Ingeo, spun from the NatureWorks polymer.^[^ ^42^ ^]^ Nowadays, the company is constructing in Thailand a 75 000‐ton annual capacity fully integrated PLA complex that involves lactic acid fermentation, lactide monomer production, and polymerization.

Polylactic acid (PLA) is a type of biodegradable and compostable thermoplastic polymer derived from renewable sources such as corn starch or hemicelluloses from wood. PLA is a biopolymer with several advantages.^[^ ^43^, ^44^, ^45^, ^46^, ^47^ ^]^ Among them, renewability, sustainability, biodegradability, compostability, and versatility—suitable for a wide range of applications such as packaging, disposable cutlery, textiles, 3D printing, and more. In addition, PLA is food safe and non‐toxic which makes it worker‐ friendly. Despite this, PLA also has some withdrawals like brittleness, hydrophilic nature, low thermal stability, low glass transition temperature and poor toughness.^[^ ^48^, ^49^, ^50^ ^]^ PLA was tested as adhesive by Bakken and Taleyarkhan^[^ ^51^ ^]^ who used crystalline and amorphous PLA as bio‐renewable green polymer adhesive for producing 2 and 3‐ply veneer plywood. The produced plywood could successfully meet the required ASTM/HVPA standards for interior use.

The aim of this study is to produce lightweight insulation boards made from kapok fibers and polylactic acid as renewable materials and therefore present a thorough evaluation in an effort to expand our options in low‐density insulation materials. Consequently, it proposes its application as a potential alternative in the regular insulation market.

## 2 Experimental Section

**Figure 1:**
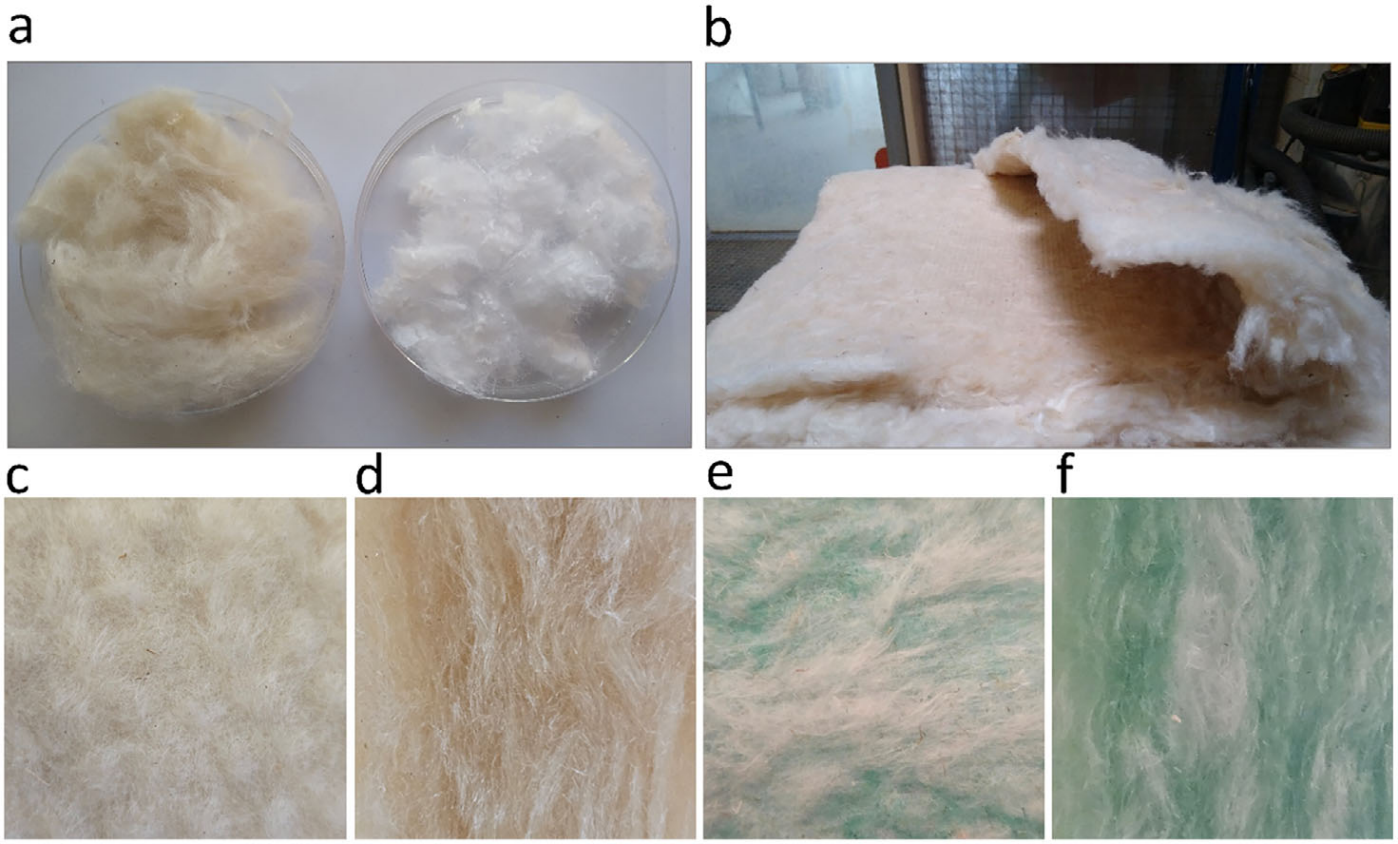
Raw material: kapok fiber (left), a) PLA (right), b) kapok‐fiber insulation boards, c) kapok‐PLA sample surface section, d) kapok‐PLA sample cross‐section, e) glass wool sample surface‐section, f) glass wool sample cross‐section.

Kapok fibers were supplied by Fränkische Schlafmanufaktur Zagekfa GmbH (Collenberg, Bavaria, Germany). Polylactic Acid (PLA) fibers (round cross section, fiber fineness 2.42 dtex, staple length 6 mm, melting point core 175 °C, melting point sheath 130 °C), and bicomponent fibers (polyethylene terephthalate and polyolefin) with a melting point core of 256 °C, and a melting point sheath of 127 °C were provided by Indorama Ventures Fibers Germany GmbH (Hattersheim, Frankfurt, Germany). Glass wool insulation material (microlite AA blankets, 0.6‐pcf) was purchased from *Johns Manville*—A Berkshire Hathaway company (**Figure** **1**). No further treatments were applied to the materials.

Lightweight natural fiber insulation boards were produced by detached kapok fibers (disaggregated by 5 bar air pressure in an atomizing nozzle) and combined with either polylactic acid (PLA) or bicomponent fiber (BF) in a rotary blending drum with an atomizing nozzle for 5 min. Boards of 65 × 65 × 2.4 cm^3^ were produced by hot air (HA) in a self‐constructed system with parts from Leister Technologies (Aachen, Germany) and MG Dampftechnik (Bedburg, Germany).^[^ ^22^, ^52^ ^]^ Boards received the hot air feed at 10 m s^−1^ by 4 min at 170 °C Lightweight natural fiber insulation boards were produced in densities of 10, 15, and 20 kg m^−3^ with a 5% of either polylactic acid (PLA) or bicomponent fiber (BF) as a binder. Boards were cooled by one day at environmental conditions without additional pressure.

Density of the boards was determined according to UNE‐EN,^[^ ^53^ ^]^ where five specimens with dimensions of 10 × 10 × 2.4 cm^3^ were used for each variable. Short‐term water absorption was developed according to UNE‐EN,^[^ ^54^ ^]^ four specimens with a density of 20 kg m^−3^and dimensions of 20×20×2.4 cm^3^ were assessed by each formulation. For compression strength, the test was developed according to UNE‐EN,^[^ ^55^ ^]^ where five specimens with dimensions 10×10×2.4 cm^3^ were assessed by variable in a universal testing machine (ZwickRoell, Ulm, Germany).

Thermal conductivity was determined from 25×25×2.4 cm^3^ samples with a density of 15 kg m^−3^, in a heat flow meter HFM 446 M, Lambda Eco‐Line (NETZSCH Group, Selb, Germany) at 10 °C, 20 °C and 30 °C. Thermal conductivity test was assessed according to UNE‐EN.^[^ ^56^ ^]^ A single specimen apparatus was used where one of the specimens is replaced by a combination of a piece of insulation and a guard plate. TGA was assessed in 10–20 mg samples placed in a Al2O3 crucible with 85 µL capacity, in TG209 F1 Iris (Netzsch, Selb, Germany) at a heating rate of 10 °C min^−1^ from 50 to 650 °C under nitrogen atmosphere. Microscopical and processed fiber images were obtained from digital microscope VHX‐7000, Keyence digital 3D reflected light microscope (Keyence, Neu‐Isenburg, Germany). To measure porosity, the samples were sectioned, and an 80X photograph was taken of the cross‐section. Subsequently, the image underwent processing, with the fibers being colored in red. In the end, the proportion of red within the entire black image was determined.

Contact angle analyzer was assessed in a drop shape analyzer (Krüss, Germany) at 20 °C by a sessile drop (drop type). A DSA25 Drop Shape Analyzer (KRÜSS, Germany) was applied to measure the static water contact angle on various substrates. The method of sessile drop was used. The volume of drop was set as 2 µL.

## 3 Results

### 3.1 Fiber Assessment

#### 3.1.1 Dimensions

**Figure 2:**
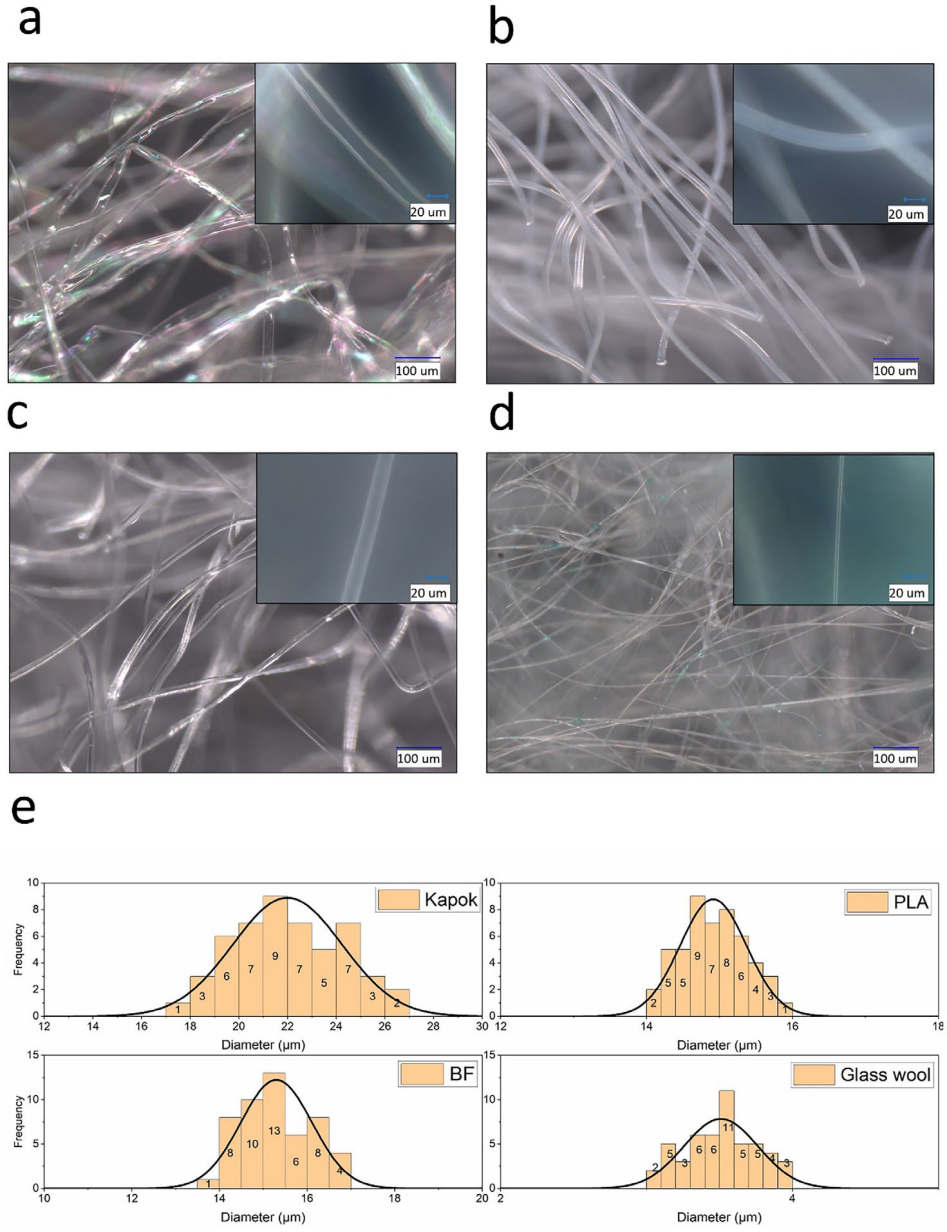
Microscopical images of a) kapok, b) PLA, c) bicomponent fibers, and d) glass wool, e) histogram of width diameter of kapok, PLA, bicomponent fiber, and glass wool.

The dimensions of kapok fiber measure approximately 21.9 (±2.24) µm in diameter, with a wall width of about 2.1 µm (**Figure** **2**), which is consistent with the data present in various literature that suggests a wall thickness ranging from 1 to 2 µm and a fiber diameter fluctuating between 15 and 35 µm.^[^ ^27^, ^57^ ^]^ The length of the fiber is about 1.13 mm^[^ ^25^ ^]^ This fiber exhibits a cylindrical shape when viewed in cross‐section and a somewhat irregular form, a characteristic shared by many natural fibers.

One distinctive feature of kapok fibers, setting them apart from other natural fibers, is their thin cell wall, which creates a channel occupying nearly 80–90% of the fiber's surface area.^[^ ^18^ ^]^ A wax coating in the cell wall, which renders the fiber highly hydrophobic, can also be observed on the kapok fiber. Kapok fibers are known for their exceptional oil‐absorption capacity.^[^ ^18^, ^27^ ^]^ The kapok fibers also have a rough fiber surface, which can prevent close contact with adjacent fibers, increasing the voids and the porosity in the material^[^ ^25^ ^]^

PLA fibers, have a diameter of around 14.9 (±0.45) µm, showcasing a cylindrical cross‐section similar to bicomponent fibers. Bicomponent fibers, which consist of a polyethylene terephthalate core (with a melting point of 256 °C) and a polyolefin sheath (with a melting point of 127 °C), exhibit irregular shapes and a circular cross‐section, measuring about 15.3 (±0.81) µm in diameter width. The diameter width of glass wool fibers, which have a straight‐line shape, is 3.5 (0.26) µm. In general, it can be also noticed a higher width variability in the diameter of natural fibers (kapok), compared with synthetic‐origin ones (glass wool).

#### 3.1.2 Thermogravimetric Analysis

**Figure 3:**
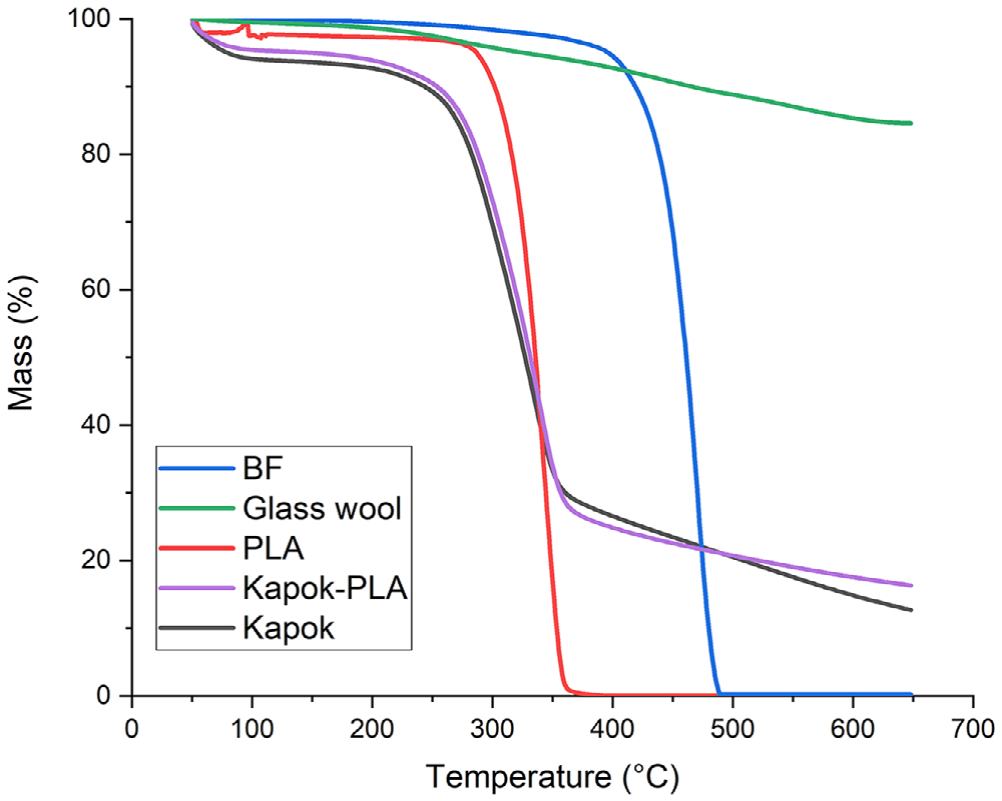
Thermal gravimetrical analysis of fibers of kapok, PLA, bicomponent fibers, glass wool, and the mixed board kapok‐PLA.

The thermogravimetric analysis (TGA) spectra are presented in **Figure** **3**. A first stage of mass‐loss takes place for lignocellulosic‐based samples between 50 and 200 °C. Therefore, this effect is larger for kapok fiber and in the mixed material kapok‐PLA because of their higher moisture content in comparison with synthetic ones or inorganic materials. This effect loss of water from lignocellulosic fiber samples as it was reported by several authors.^[^ ^58^, ^59^ ^]^ Regarded this, there are different categories of water present in natural fibers, namely free water, chemically bound water and loosely bound water. All usually are lost in the first stage of degradation.^[^ ^60^ ^]^ TGA testing reported a mass loss of 25% at, 292 °C, 320 °C and 444 °C for kapok fibers, PLA, and bicomponent fibers respectively (Figure 3). Contrasting with these values, the glass wool fiber only lost 15% of its mass up 650 °C, demonstrating that this fiber possesses outstanding heat resistance. In addition, this trend remains constant as can be seen when materials reach a mass loss of 50%, which is at 326 °C, 336 °C and 462 °C for kapok, PLA, and bicomponent fiber respectively. PLA and bicomponent fiber lost almost all its mass at 394 °C and 489 °C. It is noticeable the improvement in heat resistance of the PLA when it is mixed with kapok, in the mixed kapok‐PLA insulation boards as it can be seen in the spectra.

In another experiment, it was demonstrated that PLA could degrade at 323 °C.^[^ ^61^ ^]^ In the same procedure, it was reported a mass loss of 75% at 409 °C, 346 °C and 472 °C for kapok, PLA and bicomponent fibers respectively. The lowest thermal stability at this point is attributed again to bicomponent fibers. For composites, the addition of natural fibers to PLA decreases the mass loss, for instance at percentage weight loss of about 10%, kapok mixed with PLA composite degrades at 321 °C.^[^ ^46^, ^62^ ^]^ tested PLA and modified kapok fiber (0.5%), obtaining an initial degradation temperature of 326.5 °C (5% wt).

The PLA could be modified in order to improve its heat resistance. PLA is highly flammable and burnt with a large number of molten drops, which was attributed to the rapid degradation of molecular chains. However, with the addition of phosphorus‐nitrogenous flame retardant (NTPA), the ignition time went up to 74 s, from 51 s without it. In addition, the amount of 0.5% of kapok fiber in PLA neat inhibited heat release rate (HRR) to 657 kW m^−2^, from 766 kW m^−2^ of pure PLA. This effect is attributed to the formation of a thin char layer during the combustion, which acts as a barrier in the condensed phase. Once the flame goes through this layer, the HRR increases rapidly.^[^ ^62^ ^]^

Their value is similar even to another type of natural fiber, in this case to TGA data which indicates 50% mass loss (T50%) at 306 °C, 322 °C and 318 °C for sheep wool, goat wool and horse mane, respectively.^[^ ^63^ ^]^

### 3.2 Kapok‐Based Insulation Board

#### 3.2.1 Density and Porosity

**Figure 4:**
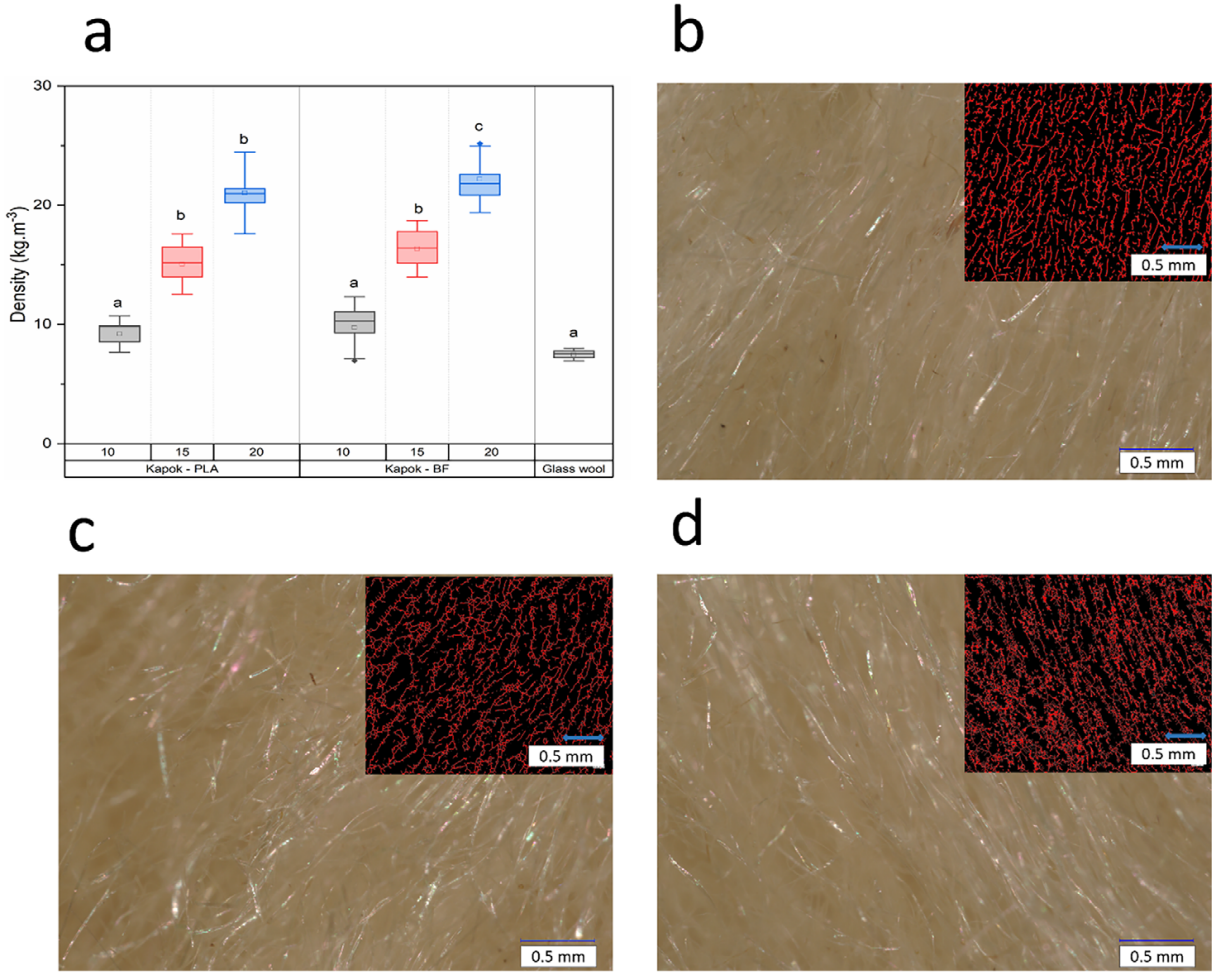
a) Densities of 10, 15, and 20 kg m^−3^ produced with kapok fiber and PLA or bicomponent fibers (BF) as a binder. Each box represents the standard deviation. Horizontal line inside the box is the median. The square represents the mean value. Letters indicate a significant different between mean values within the same material (*p* < 0.001). b–d) Microscopical image of cross‐section samples of kapok‐based boards with densities of 10, 15, and 20 kg m^−3^ and a processed image of the fibers (in red), respectively.

Density and porosity are critical properties in all fiber‐based products. Kapok‐based insulation boards are not an exception. The density of a board is mostly based on the density of the materials themselves (core material and adhesives) and the voids that the material contains. The lowest density values from commercial wood fiber insulated boards were between 50 and 100 kg m^−3^. In this work, the density of insulated boards produced with kapok fibers reached values between 10 and 20 kg m^−3^ (**Figure** **4**). These remarkable low‐values were reached for two reasons. The extreme lightness of kapok fibers, because of their high proportion between lumen and cell width,^[^ ^18^, ^27^ ^]^ and the novel production system based on hot air.^[^ ^22^, ^52^ ^]^ The length of kapok fibers was also critical to reduce the density of the insulation boards, as the length is close to 2 mm, fibers can easily form voids and therefore can reduce the density in the insulation material.

Different methods have been tested in order to reach lower densities. Lee et al.^[^ ^64^ ^]^ produced by hot‐press method (150 °C) WFIB with melamine‐formaldehyde‐urea (MFU) as a binder. Densities reached values of 100, 150, and 200 kg m^−3^. WFIB were elaborated with *Pinus radiata* (1.65 mm long and 37.2 µm diameter), fibers with the same average length of the kapok fiber. The density obtained was higher because of the use of the press method and the higher weight because of the higher diameter width of Pinus radiata fibers compared with kapok fibers, nevertheless, they have almost the same length. The same effect happened with Bayandin et al.^[^ ^40^ ^]^ and his team. They produced WFIB with a proportion of 70/30—*Pinus sibirica* wood fibers/glue mass proportion (based on the natural polymer dextran), obtaining densities of 70, 100, and 125 kg m^−3^. Insulation panels from spruce bark fibers with target densities between 160 and 300 kg m^−3^produced by wet process and without a binder, were developed by Gößwald et al.^[^ ^65^ ^]^ and his team. Spruce fiber length of 1.6 mm is similar to kapok fiber. Fuczek et al.^[^ ^1^ ^]^ produced by hot pressing, light insulation boards with Pine wood and pMDI as binding, thickness ranging from 60 to 240 mm and a reported a density of around 80 kg m^−3^. Among other natural fibers, Kuqo and Mai^[^ ^66^ ^]^ produced insulation boards with seagrass (*Posidonia oceanica* and *Zostera marina*) and pMDI as binding by cold pre‐pressed and hot‐pressed method (190 °C). The densities of the boards were between 80 and 200 kg m^−3^, a density related to the press method.

The porosity of kapok‐based insulation boards, as derived from 2‐D images, was found to be 81.18%, 77.56%, and 75.61% for densities of 10, 15, and 20 kg m^−3^, respectively (see Figure 4). These values align with the respective densities. When porosity is assessed using X‐ray (CT‐images), reported values range from 53% to 92%. De Ligne et al.^[^ ^67^ ^]^ estimated porosities of 97%, 89%, and 91% for boards with densities of 50, 160, and 140 kg m^−3^ based on their density, while the same boards showed porosities of 92%, 53%, and 80% when evaluated with X‐ray and CT images. Ali et al.^[^ ^10^ ^]^ found porosity values ranging from 18% to 20% for boards with an apparent density of 132 and 472 kg m^−3^. Sun et al.^[^ ^25^ ^]^ reached a porosity of 94% in vacuum insulation panels produced with kapok by wet process. The kapok fibers were previously cut and treated with NaOH (0.25%), drying at 105 °C and placed in a single‐side aluminized film barrier bag.

#### 3.2.2 Short‐Term Water Absorption

**Figure 5:**
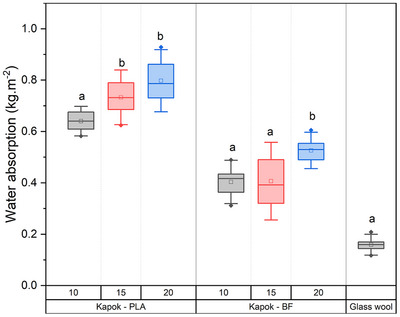
Water absorption of insulation boards with densities of 10, 15, and 20 kg m^−3^ produced with kapok fiber and PLA or bicomponent fibers (BF) as a binder. Each box represents the standard deviation. Horizontal line inside the box is the median. The square represents the mean value. Letters indicate a significant difference between mean values within the same material (*p* < 0.001).

The findings in short‐term water absorption (kg m^−2^) assessment in lightweight board samples produced from kapok and bonded with polylactic acid and bicomponent fibers possessing a density of 10, 15, and 20 kg m^−2^ are presented in **Figure** **5**. An increase in density correlates with an increase in water absorption values (kg m^−2^). This pattern is consistent in boards bonded with either polylactic acid or bicomponent fibers. Samples produced with PLA as a binder demonstrate a higher water absorption, compared with samples bonded with bicomponent fiber, indicating that PLA exhibits less hydrophobic behavior compared to bicomponent fibers in the short‐term water absorption test. Generally, all tested samples have as lower as absorption values than the commercially available insulation boards (<1 kg m^−2^). Generally, the capacity for water absorption is linked to the porosity of materials, with higher porosity usually indicating greater water absorption rates. However, this trend may not be evident, likely due to the presence of a hydrophobic wax in the kapok cell wall surface,^[^ ^18^, ^27^ ^]^ as well as the extremely low density involved, ranging between 10 and 20 kg m^−3^. De Ligne et al.^[^ ^67^ ^]^ conducted studies on a wood fiber insulation board that has a density of 50 kg m^−3^ and an estimated porosity of 97% based on its density. After 144 h, the water absorption rate was found to be 10.9 kg m^−2^. This outcome is approximately 10 times the values observed in the current experiment, suggesting a potential influence from the hydrophobic wax in kapok. Nonetheless, it is crucial to take into account the varying immersion durations. Therefore, longer immersion durations for kapok‐based boards are necessary to thoroughly investigate this aspect, being mindful not to compromise the material due to its lower density.

There is not enough information available for natural fiber insulation boards with densities lower than 20 kg m^−3^. Nonetheless, it featured a valuable discussion on the use of natural binders in insulation boards made from natural fibers. Gößwald et al.^[^ ^65^ ^]^ prepared boards with densities between 160 and 300 kg m^−3^ from natural bark fibers without additional hydrophobic additives. Water absorption values after 24 h raised up to 380%, while the thickness swelling after 24 h remains under 25%. At lower density, bark fiber boards did not show a complete wetting anymore, which goes along with a drop in water absorption down to 55% and a reduced thickness swelling. Ostendorf et al.^[^ ^68^ ^]^ tested wood fiber insulation boards produced by hot air and hot air – hot steam systems. Boards, with a density of 160 kg m^−3^ were produced from wood thermomechanical pulp (TMP) fibers provided by GUTEX, and bound with pMDI. Short‐term water absorption values from water uptake less than 1 kg m^−2^, which is the required specification for comparable GUTEX products. De Ligne et al.^[^ ^67^ ^]^ tested water absorption after 144 hours on wood fiber insulation boards with densities of 160 and 140 kg m^−3^ obtaining values of 2.5 kg m^−2^, and 5.4 kg m^−2^, respectively. It is essential to note that the production process can influence the water absorption characteristics. For instance, using a hot press can lead to an increase in density in the outer layer, thereby enhancing water absorption.^[^ ^67^ ^]^ However, in this situation, all the samples were produced using hot‐air, so this particular behavior is not observed.

Generally, it can be concluded that boards produced from kapok fibers bonded with either bicomponent fibers or polylactic acid, both of them showed closer and even lower values than commercial wood fiber insulation boards produced from thermo‐mechanical pulp fibers as Gutex multiplex‐top, Gutex ultratherm, Gutex thermoflex (<1 kg m^−2^), Steico universal, Steico special dry (<1 kg m^−2^), Pavatex pavatherm 40 (<1 kg m^−2^), best‐wood Schneider multitherm 110 (<1 kg m^−2^), and Naturheld 110 (<1 kg m^−2^). In addition, Johns Manville commercial glass wool reports water absorption values lower than 1% in volume. These water absorption results are expected, as the reached density in the present work is generally lower than in commercial lignocellulosic boards, and also because the wax layer present in the kapok fibers confer to the board a higher resistance to water absorption.

#### 3.2.3 Wettability

Wettability refers to the capacity of a material to either attract or repel water. To assess the wettability of wood fiber insulation boards, various methods are commonly employed, such as measuring the contact angle or conducting water absorption tests. These tests evaluate the ability of insulation boards to repel water and withstand the ingress of moisture, which is critical for preventing fungus decay, once those boards are in use. In the context of wood fiber insulation boards, wettability plays a significant role in determining their moisture performance. Increased wettability in wood fiber insulation boards can result in higher water absorption, increasing thermal conductivity coefficients and hence, decreased thermal insulation properties, and potential material degradation over time. Conversely, an appropriate level of water repellency is necessary to ensure the boards can effectively resist moisture and maintain their insulation performance. It is crucial to minimize water absorption and maintain good water repellency,^[^ ^69^ ^]^ and necessary to prevent moisture from permeating the insulation and causing issues like reduced thermal performance, mold growth, or structural damage.^[^ ^67^ ^]^

**Figure 6:**
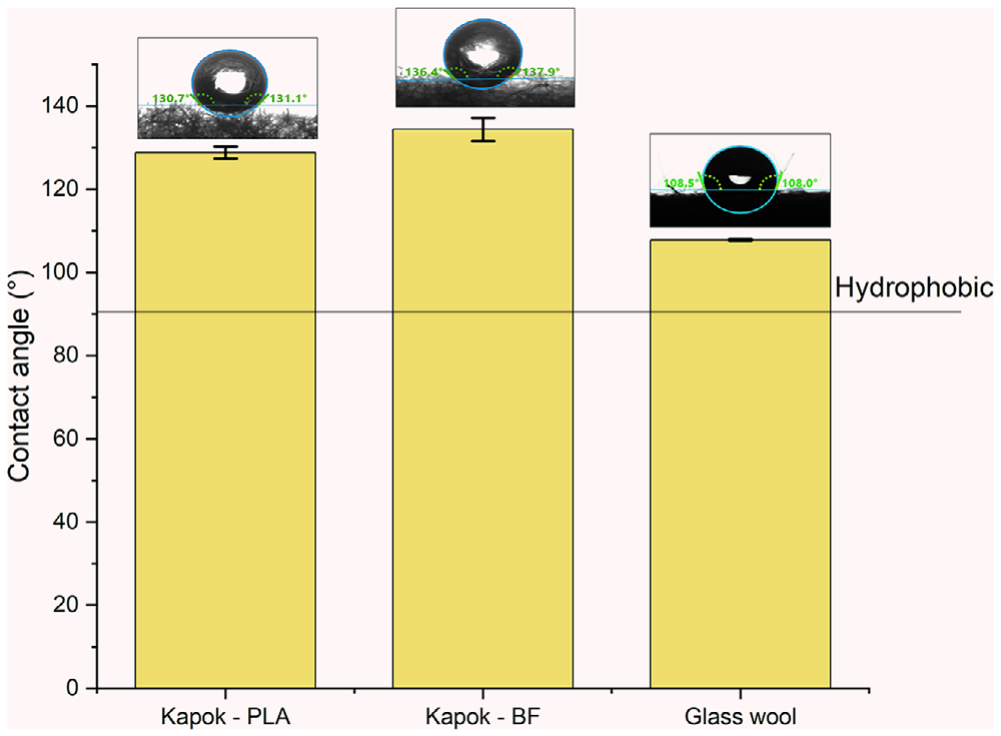
Static water contact angle of a drop over the surface of lightweight insulation boards with a density of 20 kg m^−2^ in glass wool, kapok–PLA, and kapok–bicomponent fibers (BF).

As seen in **Figure** **6**, it is evident that the panels made from kapok fibers, using polylactic acid or bicomponent fibers as a binding agent, exhibit higher angles, which implies a higher hydrophobic nature compared to the standard commercial glass wool. This observation is further substantiated by the data on water absorption rates. These outstanding values are due to the hydrophobic nature of kapok because of its wax contained in the fiber surface which increases its hydrophobicity.^[^ ^27^, ^70^ ^]^ In addition, polylactic acid also has a hydrophobic behavior. Additionally, the density and porosity of the boards also play a role in their wettability. In general, contact angle analyzer values reported high hydrophobic behavior from all samples. Chen et al.^[^ ^71^ ^]^ reported in combined monoliths of kapok and PLA without any binder a water contact angle of 141°, noticed a superior hydrophobicity behavior than the present work where a contact angle of 130° was reached.

Natural fiber insulation boards are commonly composed of natural fibers that naturally possess cellulose, a material with high affinity for water.^[^ ^72^ ^]^ These additives reduce the surface energy of the boards, making them more water resistant. In recent years, researchers have often applied hydrophobic additives or coatings to enhance the resistance of the boards to water.^[^ ^73^ ^]^ This treatment aims to minimize moisture absorption, which can have adverse effects on the insulation boards capabilities and structural integrity.

#### 3.2.4 Compression Strength

The compression strength of wood product‐based boards is a mechanical property that it is extremely related to their density, as stated by Niemz et al.^[^ ^74^ ^]^ Fiber‐based boards follow this rule too. Boards that have lower densities generally exhibit reduced compression strength values. This behavior is due to the fact that as density decreases, porosity increases, resulting in more voids within the sample, which subsequently leads to a decrease in compression strength values. There is limited detailed information available on low‐density fiber‐based insulation boards (less than 40 kg m^−3^). The compression strength of insulation boards is vital as they must resist enough pressure during transport and manipulation before their final use in construction and related uses.

**Figure 7:**
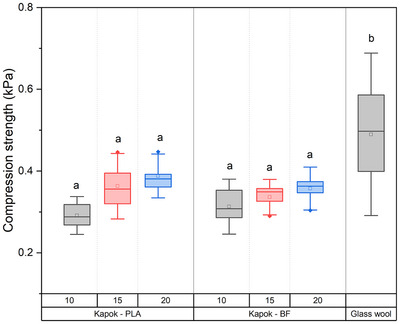
Compression strength of insulation boards with densities of 10, 15, and 20 kg m^−3^ produced with kapok fiber and PLA or bicomponent fibers (BF) as a binder. Each box represents the standard deviation. Horizontal line inside the box is the median. The square represents the mean value. Letters indicate a significant difference between mean values (*p* < 0.001).

The compression values of produced insulation boards based on kapok fibers were close to 0.4 kPa (**Figure** **7**). These compression values are extremely low compared with regular insulation boards; nevertheless, they correspond to the expected compression values of a low‐density kapok‐based insulation board, which is close to 10–15 kg m^−3^. There is no significant difference between the compression values according to the density of the boards, which is between 10, 15, and 20 kg m^−3^). The binder used in the board has no effect on compression values, as data of boards produced from polylactic acid or bicomponent fibers are statistically similar.

There is not enough information about compression values in boards with densities below 40 kg m^−3^. However, some researchers reported compression values of 17.5 and 13.9 kPa for insulation boards with a density of 80 kg m^−3^, produced with *Zostera marina* and *Posidonia oceanica* (seagrass leaves) respectively.^[^ ^66^ ^]^ The production system also could include, insulation boards produced with the same hot air system^[^ ^75^ ^]^ with bicomponent fibers as binder and birch fibers, reported a density of 100 kg m^−3^ and a compression of 6.8 kPa. Ostendorf et al.^[^ ^68^ ^]^ reported compression values between 150 and 200 kPa for wood‐fiber insulation boards WFIB produced by hot air system and hot air – hot steam system with a density between 145.57 and 168.56 of kg m^−3^. Wood source was thermo mechanical pulp (TMP) fibers provided by GUTEX, composed of Norway spruce (Picea abies Karst.) and silver fir (Abies alba Mill.) at a mixing ratio of 80 to 20%. Although the length of Norway Spruce is between 1.7 to 3.7 mm and the width is between 21 and 40 µm, the proportion between lumen and cell wall is not like kapok, hence the density obtained was higher.

Xu et al.^[^ ^62^ ^]^ tested PLA neat with modified kapok fibers (0.5%). Their team reported that the homogeneously dispersed lightweight kapok fibers enabled the impact strength by 18% compared with pure neat PLA. However, as modified kapok fibers increased, tensile and flexural strength decreased. These researchers observed that the incorporation of fiber‐like materials gave rise to an improvement in toughness at the sacrifice of the tensile strength of PLA composites.

#### 3.2.5 Thermal Conductivity

Thermal conductivity *λ* defines the steady state heat flow passing through a unit area of a homogeneous material, 1 m thick, induced by a 1 K difference of temperature on its faces. It is expressed in W m^−1^ K^−1^ and it is measured in compliance with EN 12664 (low thermal resistance). The thermal conductivity could be affected by humidity and temperature fluctuations, moisture, freeze/thaw cycles, UV radiation, and so on.^[^ ^76^ ^]^ The target in insulation boards is to have the lowest thermal conductivity possible. The density has a direct relation with thermal conductivity, as with increasing density, also the thermal conductivity will increase.^[^ ^77^, ^78^ ^]^ However, there is an important phenomenon, related to low density and thermal conductivity in WFIB. On the other hand, the porosity has an inverse effect in the thermal conductivity. As higher the porosity, the thermal conductivity value diminished. The porosity of fiber insulation materials are between 82 and 97% (based on density) and 53–92% (based on X‐ray CT‐images).^[^ ^67^ ^]^ Normally, as density decreases, also the thermal conductivity decreases, but this effect holds only until some point which is between about 50 and 80 kg m^−3^, after this point, the trend changes. A critical factor for thermal conductivity is also the porosity of the material.^[^ ^79^ ^]^ In fibrous materials, like lignocellulosic‐fiber insulation materials the heat could be transfer by three ways; conduction through direct contact between fibers, radiation between fibers, and convection in the voids between fibers.^[^ ^80^ ^]^ In a similar way, it is also postulated that heat can be transferred through a solid structure via lattice vibrations, also known as phonons, involving the interactions between atoms and molecules, as well as chemical bonds. Another method for heat transfer is through gas particles, where heat is passed from molecule to molecule due to the collisions among gas components. A third method involves heat radiation, which is the emission of electromagnetic radiation by the surface of a material within the appropriate infrared range. Finally, there is gas convection, which is particularly significant in insulation boards and involves the movement of air and moisture. The effectiveness of convection is influenced by temperature and pressure; as the density increases (meaning porosity decreases), convection can become negligible.^[^ ^76^ ^]^

At a temperature of 26 °C, polylactic acid has a thermal conductivity approximately equal to 0.13 W m^−1^ K^−1^.^[^ ^81^ ^]^ Meanwhile, kapok fibers have a documented thermal conductivity of 0.042 W m^−1^ K^−1^ when measured at 33.4 °C,^[^ ^82^ ^]^ and between 0.03 and 0.04 W m^−1^ K^−1^ for densities between 5 and 40 kg m^−3^.^[^ ^83^ ^]^ Among the binders, it is important to mention that polylactic acid itself does not have high melting temperature resistance, on the contrary, it shows high flammability and brittleness.^[^ ^45^, ^49^ ^]^

**Figure 8:**
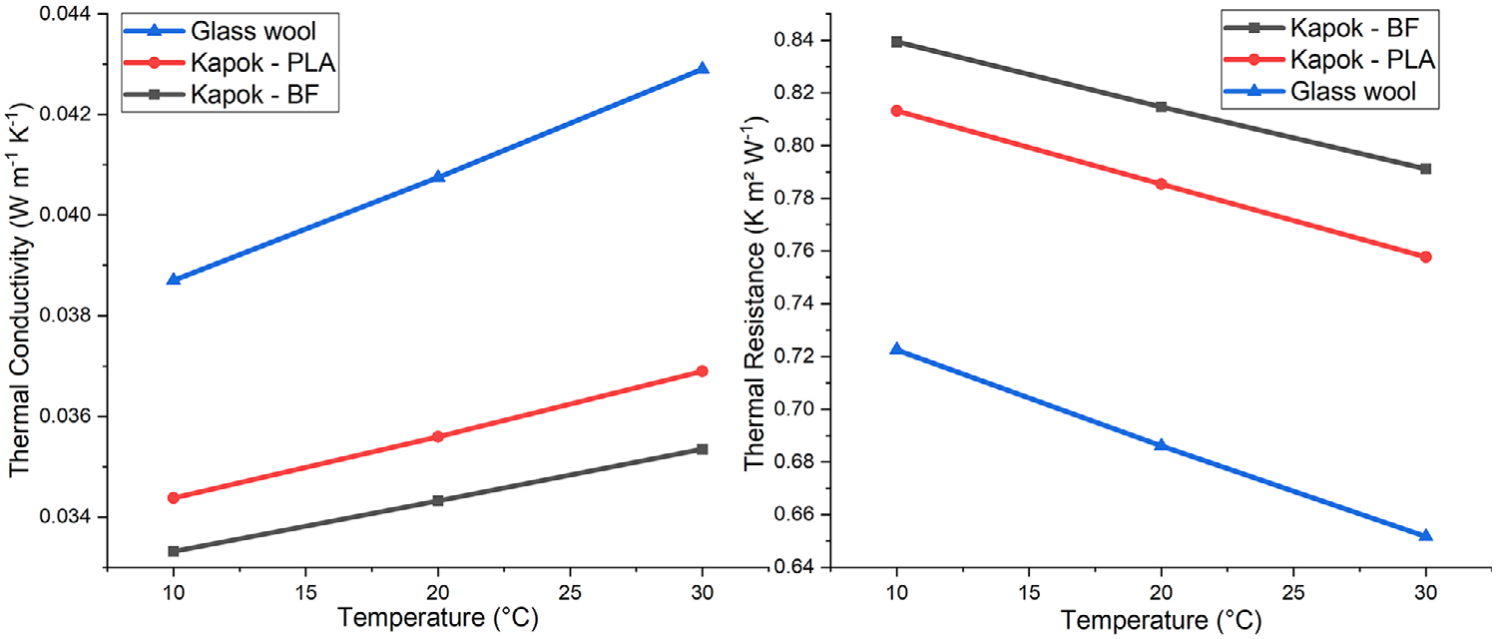
Thermal conductivity and thermal resistance values of boards produced with kapok fibers with PLA and bicomponent fiber (BF), and glass wool.

Thermal conductivity of kapok fiber boards of 15 kg m^−3^ density bound with polylactic acid (PLA) and bicomponent fibers at 20 °C was 0.03560 W m^−1^ K^−1^ and 0.03432 W m^−1^ K^−1^, respectively (**Figure** **8**). The thermal conductivity value of commercial glass wool reached 0.04075 W m^−1^ K^−1^ at 20 °C, a higher value that the renewable fiber‐based board produced in the present work. One reason is the high ratio between the kapok wall and lumen, which provides a large volume of kapok lumen fibers capable of retaining significant amounts of air inside,^[^ ^18^, ^27^ ^]^ giving to the kapok‐boards high thermal resistance. Thermal conductivity values reached are in general lower than usual ones, reported for natural fiber‐based and commercial non‐renewable insulation boards.^[^ ^7^, ^44^, ^50^ ^]^ Barkhad et al.^[^ ^84^ ^]^ and his team reported thermal conductivity values of 0.0682 W m^−1^ K^−1^ in boards produced with 60% PLA and 40% date pit powder. Another instance is the study by,^[^ ^59^ ^]^ which developed a thermal insulating material using Eucalyptus globulus leaves and wheat straw fibers. They found that the average thermal conductivity of the dried leaves ranged from 0.045 to 0.055 W m^−1^ K^−1^ for temperatures between 10 and 60 °C. The hybrid specimens reached an upper thermal conductivity value of approximately 0.065 W m^−1^ K^−1^ at the highest temperature of 60 °C. In the same line,^[^ ^58^ ^]^ developed five hybrid samples using surface fibers from date palm trees (PTSF) and apple of sodom fibers (AOSF) in varying mass and density ratios, utilizing wood adhesive as a binder. Thermal conductivity was measured across a temperature range of 10 °C to 50 °C, yielding average values between 0.04234 and 0.05291 W m^−1^ K^−1^.

Xu et al.^[^ ^62^ ^]^ and his team, modified the kapok fiber surface by adding NaOH to reduce the wax on the surface, then dried, blended and extruded with other materials in order to obtain a PLA matrix containing 0.5% of kapok fiber. Their team also added phosphorus‐nitrogenous flame retardant (NTPA).

## 4 Conclusion

Low‐density insulation boards of 10, 15, and 20 kg m^−3^, were produced from kapok fibers and bound either using polylactic acid or bicomponent fibers, founding them suitable for future industry applications. This suitability stems from their lower thermal conductivity and water absorption rates compared to commercial options. Regarding compression values, density seemed to have no effect, likely due to the extremely low‐density values. Water absorption showed a direct link with density, a pattern confirmed by absorption and wettability tests. These values could potentially be considered lower since they match the commercial board values. In terms of thermal conductivity, the boards produced exhibited encouraging results by achieving values lower than those of commercially available ones highlighting the tentative scaling‐up of the kapok‐based produced boards.

## Conflict of Interest

The authors declare no conflict of interest.

## Data Availability

The data that support the findings of this study are available from the corresponding author upon reasonable request.
